# Individual response variations in scaffold-guided bone regeneration are determined by independent strain- and injury-induced mechanisms

**DOI:** 10.1016/j.biomaterials.2018.11.026

**Published:** 2019-02

**Authors:** Natalie Reznikov, Oliver R. Boughton, Shaaz Ghouse, Anne E. Weston, Lucy Collinson, Gordon W. Blunn, Jonathan R.T. Jeffers, Justin P. Cobb, Molly M. Stevens

**Affiliations:** a Department of Materials, Department of Bioengineering and Institute for Biomedical Engineering, Faculty of Engineering, Imperial College London, United Kingdom; b Department of Surgery and Cancer, Faculty of Medicine, Imperial College London, United Kingdom; c Department of Mechanical Engineering, Faculty of Engineering, Imperial College London, United Kingdom; d The Francis Crick Institute, United Kingdom; e Department of Materials and Tissue, Faculty of Medical Sciences, University College London, United Kingdom

**Keywords:** Bone regeneration, Scaffold, Ovine model, Stiffness, Strain, microCT

## Abstract

This study explored the regenerative osteogenic response in the distal femur of sheep using scaffolds having stiffness values within, and above and below, the range of trabecular bone apparent modulus. Scaffolds 3D-printed from stiff titanium and compliant polyamide were implanted into a cylindrical metaphyseal defect 15 × 15 mm. After six weeks, bone ingrowth varied between 7 and 21% of the scaffold pore volume and this was generally inversely proportional to scaffold stiffness. The individual reparative response considerably varied among the animals, which could be divided into weak and strong responders. Notably, bone regeneration specifically within the interior of the scaffold was inversely proportional to scaffold stiffness and was strain-driven in strongly-responding animals. Conversely, bone regeneration at the periphery of the defect was injury-driven and equal in all scaffolds and in all strongly- and weakly-responding animals. The observation of the strain-driven response in some, but not all, animals highlights that scaffold compliance is desirable for triggering host bone regeneration, but scaffold permanence is important for the load-bearing, structural role of the bone-replacing device. Indeed, scaffolds may benefit from being nonresorbable and mechanically reliable for those unforeseeable cases of weakly responding recipients.

## 1 Introduction

Bone as a tissue is generally capable of complete, traceless healing following trauma [1]. However the two global medical device industries of joint replacement and dental implants are not yet universally successful [2], resulting in a rampant rate of revision surgeries associated with large areas of bone loss [3], and generating a potentially enormous demand for bone grafts [4]. In the case of small and/or confined defects, autologous bone transplant is the unparalleled gold standard, and there are additional choices for filling such defects using synthetic, predominantly inorganic compounds. The situation is different where large defects require reconstruction. When a load-bearing “prosthetic structure”, rather than a filler, is necessary, surgeons do not yet have an ideal solution. Although strength and rigidity of orthopaedic devices are obviously crucial, when a too stiff orthopaedic implant is installed, the bone around the implant is shielded from the load [5]. Under these new mechanical conditions of reduced load, an imbalanced remodeling begins, making the host bone thinner and more porous. Eventually, stiff metal devices or prostheses, whose apparent moduli are mismatched with the host bone, often lead to additional and more extensive revision surgery [6]. Porous metallic devices better approximate the apparent elastic modulus of bone [[7], [8], [9]] but relatively little variability is offered amongst these devices to address the varying mechanical demands of different parts of the skeleton and varying biological profile, activity level and regenerative potential of the recipients. By avoiding strength and rigidity overdesign of orthopaedic implants and approximating their stiffness to bone stiffness, stress shielding may be reduced [[10], [11], [12]], and adequate and physiological stimulation of the host bone remodeling may be achieved [13].

At the tissue and cells scale, the requirements for bone regeneration-guiding scaffolds have been summarized [14]: Successful regeneration of bone requires sufficient rigidity for mechanical support and stability, and at the same time sufficient compliance for mechanical stimulation [15,16]. Scaffolds have to be porous enough to allow tissue ingrowth and vascularization but not so porous that structural integrity is lost [[17], [18], [19]]. An ideal scaffold should be temporary and eventually degrade, but not before it is replaced with mature regenerated host bone having adequate mechanical properties [20]. Scaffolds should ideally be osteoinductive (*i.e*., evoking nascent bone formation) [21], but be able to compartmentalize osteogenesis within a targeted repair site. To summarize, repair of large bone defects with artificial constructs capable of both i) fulfilling a prosthetic, structural function, and ii) guiding host bone regeneration must address the multidimensional Goldilocks’ principle of having “not too much and not too little”.

Being the site of most joint replacement revision surgeries, the metaphysis is an anatomic and functional site of high clinical relevance, especially in the aftermath of failed joint replacement. In comparison with the diaphyseal compact bone that is perfectly adapted to bending and torsion (as evident from the “hollow tube” layout of the diaphysis [22]), trabecular bone of the metaphysis satisfies a distinct set of biomechanical requirements. These requirements include counteracting multidirectional loads that occur during movement, effectively transferring load to the shaft, and creating a shock-absorbing interphase between the relatively low modulus cartilage and the stiff and solid compact bone of the shaft [23]. For this reason, even in such intensively loaded locations such as the knee or hip joints, in large and physically active organisms, trabecular bone of the metaphyses always remains porous and reticulate and cannot be replaced by solid and stiff compact bone. This study is a proof-of-concept, testing the optimal structural and mechanical properties of an artificial porous structure that allow the emulation of the multidirectional load transfer behaviour of metaphyseal bone, while also providing mechanically-driven scaffold integration and host bone regeneration.

The large animal (sheep) experimental model has the advantage of a bone loading magnitude and mode that approximate the conditions of bone loading in humans [24,25], and the benefit of genetic diversity and individual variation that makes them closer to the human population than inbred lines of rodents [26]. In order to evaluate a broad range of scaffold apparent moduli and their effect on bone regeneration in a large animal model, we used two substrates having high or low material moduli (titanium or polyamide, respectively), and we further varied apparent modulus values by using different scaffold architectures [27,28] via selective laser sintering [29]. The resultant apparent moduli of the scaffolds were matched to the normal high and low values of trabecular bone modulus: close to the apparent modulus of human trabecular bone in the proximal femur [30] (titanium biomimetic, TB), and close to the apparent modulus of human trabecular bone in the vertebrae [31] (polyamide biomimetic, PB). We also refer to these designs as biomimetic because the connected elements are triangulated rendering the entire structure more stable in a vast range of loading directions [32]. Control structures were designed to match the material modulus of compact bone [33] (most stiff titanium control, TC), and to be below the modulus of trabecular bone (polyamide control, PC, with cubic symmetry, which is stable only in 3 principal loading direction and is compliant and easily deformable in all non-axial directions of loading) (Table 1). Since the scaffolds were designed to locally replace porous trabecular bone, complete pore occlusion and bone solidification were not anticipated. Rather, attainment of mature bone microstructure was sought after.

**Table 1:** Scaffolds used in the study in decreasing order of apparent modulus.

Scaffold	Titanium control	Titanium biomimetic	Polyamide biomimetic	Polyamide control
Acronym	TC	TB	PB	PC
Material modulus	100 GPa	100 GPa	2 GPa	2 GPa
Apparent modulus	7100 MPa max:min = 1.5	1400 MPa max:min = 1	220 MPa max:min = 1.5	220 MPa max:min = 5.3
Stiffness in comparison to trabecular bone	Ten-fold higher	Matched	Matched to lower range, isotropic	Matched to lower range, anisotropic
Design	Octetruss	Stochastic	Octetruss	Orthogonal
Prototype				

## 2 Materials and methods

Four types of cell-free porous scaffolds of different stiffness (six replicas of each), were inserted into the distal femurs of 12 ewes (Fig. S1). The animal experiment complied with the ARRIVE guidelines and was carried out in accordance with the U.K. Animals (Scientific Procedures) Act, 1986. Each animal received two different scaffolds (one per each femur). The properties of the scaffolds are summarized in Table 1. We evaluated the total amount of bone tissue ingrowth as a percentage of the defect volume not occupied by the scaffold.

### 2.1 Scaffold design, fabrication and preparation

Uniformity of stress within and around an implanted scaffold can be controlled by the scaffold design. For example, a rectangle-based structure will have a high stiffness in the orthogonal directions coinciding with the directions of its elements, but it will have low stiffness in all other directions because non-axial loading would result in bending and shear. In contrast, a triangle-based structure performs isotropically in all directions of loading because triangulated elements work collectively in axial loading [32,34]. Avoiding shear and bending at the structural level renders triangulated structures advantageous for the conditions of multidirectional loading [35].

Four truss scaffolds were designed in Rhinoceros 3D software (McNeel Europe, Barcelona, Spain) to be fabricated using selective laser sintering.

1. A polyamide (PA12, nylon) scaffold was designed using octetruss space-filling elements [35]. Each octetruss comprises one dodecahedron with 6 internal axes and two tetrahedrons with four internal axes each. This design was fully triangulated in order to reduce shear and bending stresses amongst the struts and compensate for the relatively low elastic modulus of the material used (around 2 GPa). Nominal porosity in this scaffold was 0.45. This scaffold will be referred to as the Polyamide Biomimetic Scaffold, PB.
2. A stochastic commercially-pure titanium scaffold was designed by filling a 3D volume with a random distribution of about 5000 points using a Poisson disk algorithm and connecting each point with its nearest neighbors to achieve a desired connectivity (average connectivity of 4.5) as described in Ref. [36]. Thickness of these connections was locally altered so the stiffness of the produced structure [17] would be equal to the stiffness of the bone it would be replacing. Sheep bone material properties and stiffness data were acquired through a combination of CT-scanning and mechanical testing of ex-vivo specimens and from the literature [18,19]. This scaffold will be referred to as the Titanium Biomimetic Scaffold, TB. Nominal porosity in this scaffold was 0.8, averaged for the entire scaffold.
The two control scaffolds were designed as follows: One made of the same polyamide material but with a different design to assess the effect of scaffold architecture on bone ingrowth, and one made out of titanium but with the same design as the Polyamide Biomimetic Scaffold to assess the effect of scaffold material on bone ingrowth.
3. A polyamide (PA12, nylon) scaffold was designed with a cuboid as the space-filling element. In this scaffold, all the struts connected at right angles and essentially formed a 3D orthogonal grid. Such a structure is stiff when loaded in three orthogonal directions that coincide with the orientations of the struts. However, when loaded in all other possible orientations, this structure is at risk to yield in shear because each space-filling element (i.e., each cubic unit) deforms towards a rhomboid. Nominal porosity in this scaffold was 0.35. This scaffold will be referred to as the Polyamide Control Scaffold, PC.
4. A commercially pure titanium scaffold was produced using the same octetruss space filling elements as the Polyamide Biomimetic, with the dimensions of the unit cell slightly scaled up due to the metal sintering restriction. Nominal porosity in this scaffold was 0.6. The material modulus of titanium exceeds that of polyamide 12 by two orders of magnitude. Therefore, this scaffold served as a high stiffness control item and will be referred to as Titanium Control Scaffold, TC.

The polyamide biomimetic and control scaffolds were manufactured using selective laser sintering using an EOS FORMIGA P110 machine (Electro Optical Systems EOS Ltd., Warwick, UK). After production, the items were removed from powder substrate, cleaned by air blasting, water jet blasting and then washed by hand. Steam sterilization was performed in an autoclave at 121 °C for 20 min (Prestige Medical Portable Autoclave, Fisher Scientific UK, Loughborough, UK) in sealed autoclave bags.

The titanium scaffolds were manufactured on a Renishaw AM250, a metal powder bed fusion system, onto a titanium substrate. The workings of the system have been described previously in Ref. [28]. Commercially Pure Titanium grade 2 (CP-Ti) powder was used having a particle size range of 10–45 μm (D50: ∼27 μm). After production, items were removed from the powder substrate by electro discharge machining and shot-blast. Specimens were rinsed and cleaned ultrasonically in a cleaning solution (0.2 μm filtered water and Decon Neutracon) followed by sterile isopropanol to remove all contaminants (Hunt Developments UK Ltd). Following filtered drying, specimens were vacuum packed/sealed in pouches and sterilized via Gamma Irradiation (25–35 kGy).

All cylindrical samples were tested mechanically along the cylinder axis according to ISO 13314:2011 (quasi-static compression test of porous structures), using a materials testing machine (Instron 8872) and a 1, 5 or 25 kN load cell depending on the specimen. Specimens were crushed at a constant strain rate of 2 mm/min (∼0.1 strain/min). Displacement between the compression platens was measured by linear variable differential transformers (LVDTs) and recorded at 30 Hz. Strain was calculated as the LVDT displacement divided by the specimen height (nominally 15 mm), and stress was the measured load divided by the specimen's initial cross-sectional area (nominally 200 mm^2^). A preliminary sample of each type was compressed to 50% strain to estimate the yield strength. To determine the mechanical properties per unique scaffold (n = 5), samples were loaded to 50% strain with a hysteresis loop between 70% and 20% of the estimated yield strength to account for the localized plasticity in porous materials which reduces the slope of the initial loading curve. Stress-strain curves were recorded and elastic modulus was calculated as the linear regression of the hysteresis loop.

The mechanical testing was used to validate FEA models of the scaffold prototypes in Karamba3D (a parametric structural engineering tool which provides accurate analysis of spatial trusses, frames and shells, embedded in Rhinoceros 3D design software). Multidirectional loading simulation was performed with increments of 15° of the φ and θ angles. The ratio of the minimal and the maximal apparent modulus values was recorded as the anisotropy coefficient in order to reflect the realistic loading environment of the implants in the knee joint which is subjected to movement. The average of the moduli measured at the various angles of loading was calculated as well. In summary, the scaffolds can be ranked by increasing stiffness as PC, PB, TB, and TC, and their moduli, anisotropy coefficients and design snapshots are shown in Table 1.

### 2.2 Surgical procedure

Twelve skeletally mature (older than 4 years) non-pregnant female ewes (breed mule), were enrolled in the study. Animals’ weights varied between 62 and 85 kg (mean weight 72.8 kg, SD 5.7 kg). Ethical approval for this study was received from the United Kingdom Home Office (Project License Number 70/8247). Both hind legs of each sheep were used to create metaphyseal bone defects, diameter 15 mm, depth 15 mm. One scaffold (diameter 16 mm, length 15 mm) was inserted into each hind leg, in the distal medial femoral condyle. The radial oversizing of the scaffolds was planned based on the mock surgery experiments where loose fit was observed when the master drill diameter and the scaffold diameter were both 15 mm. Such extent of oversizing (or press-fit) is a common practice in orthopaedic surgery and is helpful for implant stabilization within trabecular bone [37]. The scaffolds were allocated to the sheep randomly, as displayed in Fig. S1. In total, 24 scaffolds, 6 of each kind, were assigned.

Pre-operative antibiotics were given to each sheep and continued for 3 days postoperatively (Cefalexin 1ml/25 kg animal once a day). Under general anaesthesia the distal medial femoral condyle was exposed and periosteum removed over the area of implant insertion. A cylindrical defect was created by sequential using of drills of increasing diameter, equipped with a depth gauge (5 mm, 8 mm, 10 mm, 12 mm, 14 mm and 15 mm) perpendicular to the bone surface. At the end of this, a 15 mm reamer was used to ensure an evenly shaped cavity and a flatter base of the cavity.

Simultaneously with the defect preparation, a scaffold was centrifuged in 30 ml of animal's own blood (drawn from the jugular vein) in a sterile test tube at 3000 revolutions per minute for 3 min. This step was conducted in order to dislodge micrometer-scale air bubbles from the rough laser-sintered surface of the scaffold. The centrifuged scaffold was press fit into the cavity by hand, followed by gentle application of force from a surgical mallet. The wound was closed in 3 layers (fascia, subcutaneous soft tissue and then skin) using resorbable sutures. The wound area was sprayed by an aseptic spray (Opsite). Postoperatively, once a swallowing reflex had been established the animal was transferred back to a single pen with straw bedding. The animals recovered unrestrained, in sternal recumbency and were allowed hay, concentrate and water as is the normal feeding regime. The sheep received analgesia for 60 h post-operatively (Fentanyl 75 mcg patches).

Recovery was uneventful in all but one animal who was limping on the left leg for 5 days (animal 5, Titanium Biomimetic Scaffold). Plain radiographs of the left hind leg were unremarkable. The animal recovered spontaneously, with no further evidence of an abnormal gait or discomfort for the rest of the study period. The animals’ care was in accordance with institutional guidelines.

Oxytetracycline was administered by slow intravenous injection on day 28 postoperatively at 30 mg per kg of body weight in order to tag the site of active osteogenesis with a fluorophore and to quantify the rate of bone accretion over the last two weeks of the in-life phase.

All animals were euthanized at 6 weeks post-operatively. The femur was disarticulated and its distal metaphysis was cut and trimmed to the size of about 5 cm in all dimensions. The samples were stored in formalin until the analysis and characterization procedure in labeled glass jars at room temperature. Scaffold placement and retrieval are illustrated in Fig. S1.

### 2.3 Micro-computed tomography (micro-CT)

The trimmed samples were lightly blotted of formalin, wrapped watertight in a nitrile glove to prevent desiccation and were mounted on a cylindrical specimen holder with tape. The mounting was done with the longitudinal axis of the scaffold oriented vertically and the medial surface of the condyle facing the holder. Tomographic imaging was conducted in an Xradia Versa 510 (Zeiss) at 140 kV/10 W or 80 kV/7 W, with 3201 projections and a pixel size between 27 and 34 μm. The 3D reconstructed tomographic images were segmented using Amira 5.3.2 (FEI) and analyzed using ImageJ/BoneJ software [38]; to account for the possibility of segmentation bias, the segmentation was reproduced using Dragonfly image analysis software (Object Research Systems Inc.). Segmentation of the sample contained 2 steps for the polyamide items and 3 steps for the titanium items. In the case of the polyamide items, in which the scaffold material had the same radiographic density as soft tissue, the defect configuration was labeled based on its actual size and then the ingrown bone was labeled within the defect only based on the global threshold. The size of the defect was calculated in pixels, the scaffold porosity (as a fraction of unity) was known by design and the bone volume in pixels was divided by the product of the defect volume and the scaffold porosity. The CT scans of the titanium specimens revealed beam hardening, visible in a tomogram as a structureless halo of higher pixel values around the metal elements. The defect was delineated as a cylindrical volume, within which the scaffold material was labeled based on the pixel value followed by dilate operation, kernel size 3 pixels. Then the ingrown bone material within the defect volume but outside the scaffold volume was labeled based on its local gray value in every second 2D slice using local thresholding and then the bone label was interpolated to cover all 2D slices of the tomogram. This multi-step labeling of bone based on the local gray values was necessary to avoid the inclusion of bright pixels around the metal elements that were not bone, but the effect of beam hardening. Three-dimensional rendering and segmentation are illustrated in Fig. 2.

For the assessment of peripheral and central osteogenesis in the defect a library was created for independent scoring. It contained 2D images of 5 virtual sections for each specimen, perpendicular to the central axis of the cylindrical scaffold, taken at depths of 2, 5, 8, 11 and 14 mm from the periosteal surface. The peripheral and central osteogenesis were scored on each image using a scale from 0 to 4 by three operators independently, using a shared list of criteria. The results were compared and in case the inter-operator score discrepancy exceeded 1 unit (one scaffold, peripheral ingrowth), the images were rescored until a consensus was reached. Scoring was not blinded because the scaffold types were easily identifiable in the original images.

Data analysis was performed using IBM Statistics v24 (IBM, USA). Wilcoxon Signed Rank tests (for non-parametric data) were performed to compare bone ingrowth into the four different scaffolds. The predicted inverse correlation between scaffold stiffness and bone ingrowth across all the samples was assessed using least squares regression. For this regression, data were first logarithmically transformed due to the scaffold stiffness scale being exponential (ranging from 200 MPa for the most compliant scaffold up to 7000 MPa for the stiffest scaffold). P values less than 0.05 were deemed to be statistically significant.

### 2.4 Microscopy

Following tomographic imaging, the samples were processed for microscopy.

#### 2.4.1 Sample embedding and sectioning

Wet and fixed samples were dehydrated for embedding by immersing in methylated spirits of increasing concentration (50%, 75%, 85%, 95% and 2 × 100%). 100% spirit was replaced by chloroform for 24 h, and then the medium was substituted by methylmethacrylate resin (LR White) for 72 h. Following resin saturation, the accelerator was added and the blocks were polymerized at low temperature (−20 °C).

The blocks were trimmed and mounted for sectioning using a water-cooled bandsaw (Exakt 311, Exakt, Germany). The block was cut parallel to the surface of the medial condyle and, therefore, parallel to the scaffold face.

For the first two animals in the study, the central section of each specimen was further processed for electron microscopy and high resolution μCT.

For all the samples one central slice was prepared for microscopy. Samples were mounted and grinding was performed down to sample thickness of approximately 50 μm by successive silicon carbide grinding cloths, followed by polishing with an alumina suspension (Exakt 400 CS Grinder, Exakt, Germany). These sections then underwent fluorescent microscopy, followed by histological staining.

#### 2.4.2 Fluorescent microscopy

Microscopy was performed using a Zeiss Wide-Field light microscope (WF3 Zeiss Axio Observer, Zeiss Germany). The 5× objective was used and the Zen Pro software program (Zeiss, Germany) was used to select the oxytetracycline emission wavelength of 547 nm and Brightfield channels. Tiled microscopic images were recorded across the whole slide using the Zen Pro auto-stitching function.

#### 2.4.3 Staining and light microscopy

Toluidine blue was placed on the sections after fluorescent microscopy. It was left on for 20 min and then washed off, followed by 20 min of Paragon stain. Microscopy was performed in the bright-field mode (WF3 Zeiss Axio Observer, Zeiss Germany). Tiles of microscopic images were recorded and then auto-stitched together using Zen Pro software (Zeiss, Germany).

#### 2.4.4 Electron microscopy imaging

Explants from the animals 1 and 2 (four bone samples containing four different scaffolds) were used for Scanning Electron Microscopy imaging. After resin embedding as described above, one section of 1 mm thickness was polished with a series of grinding papers but not thinned. These thick embedded undemineralized polished sections were mounted on standard EM holders with silver paint and coated with carbon to improve surface conductivity. These samples were imaged in LEO Gemini 1525 FEG-SEM (Zeiss, Oberkochen, Germany) at 7 kV, working distance 10 mm and simultaneous acquisition of the image in secondary electrons mode (SESI detector) and backscattered electrons mode (ESB detector) in order to obtain both topographic and compositional information of the same area of interest.

#### 2.4.5 High-resolution micro-CT

For high resolution micro-CT 1 mm thick rods were machined from the embedded bone samples (the same explants as those used for electron microscopy imaging). These rods containing fragments of the scaffold struts alternating with resin were mounted vertically in Xradia Versa 510 (Zeiss) and scanned at a resolution 20 μm per pixel, 40 kV, 75 μA, 400 projections per scan, to localize suitable areas of bone ingrowth within the scaffold pores. Following the survey scan and localizing the coordinates of the small area of interest, a high-resolution scan was performed at 40 kV, 75 μA, 3200 projections and pixel size 0.4–0.5 μm in order to visualize the type of nascent bone and vestiges of associated soft tissue.

## 3 Results

### 3.1 Overall effect of scaffold stiffness on bone regeneration

**Fig. 1:**
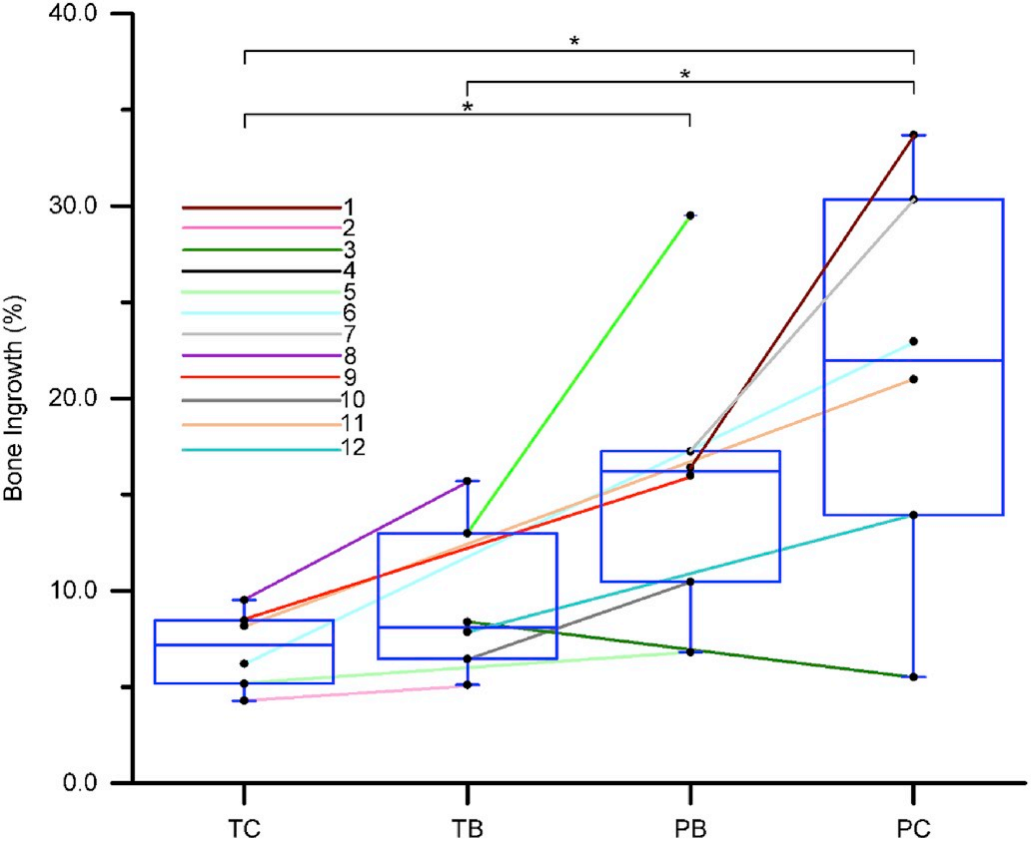
Bone ingrowth observed in the four scaffold groups (Titanium control TC, Titanium biomimetic TB, Polyamide biomimetic PB and Polyamide control PC), 6 measurements per group. Individual measurements obtained from μCT in 3D are plotted and those originating the same animal (from left and right femurs) are connected with a line, between and across the 4 groups reflecting the implant allocation (lines colored according to the animal number). Samples are plotted in the order of decreasing stiffness: TC – stiff, TB and PB – matching the moduli of trabecular bone, PC – most compliant and anisotropic. Box plots are superimposed onto the same graph. Statistically significant differences are indicated by an asterisk (*p* < 0.05, Wilcoxon Signed Rank Test, *n* = 6).

Total bone ingrowth tended to increase as scaffold stiffness decreases (Table 2, Fig. 1). There was a moderate, inverse correlation between scaffold stiffness and bone ingrowth across all the samples (*r*^2^ = 0.419, *p* = 0.001) as shown in Fig. S2.

**Table 2:**
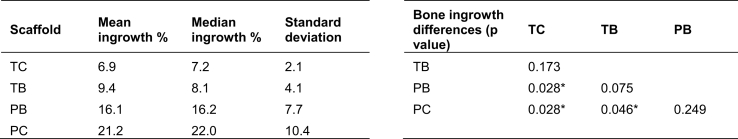
Bone ingrowth into the four different scaffolds shown in the left table. *p* values for differences between ingrowth amongst the scaffold are shown in the right table with asterisks indicating statistically significant differences (*p* < 0.05, Wilcoxon Signed Rank Test, *n* = 6). TC – titanium control, TB – titanium biomimetic, PB – polyamide biomimetic, PC – polyamide control. Measurements are based on μCT and are done in 3D.

**Fig. 2:**
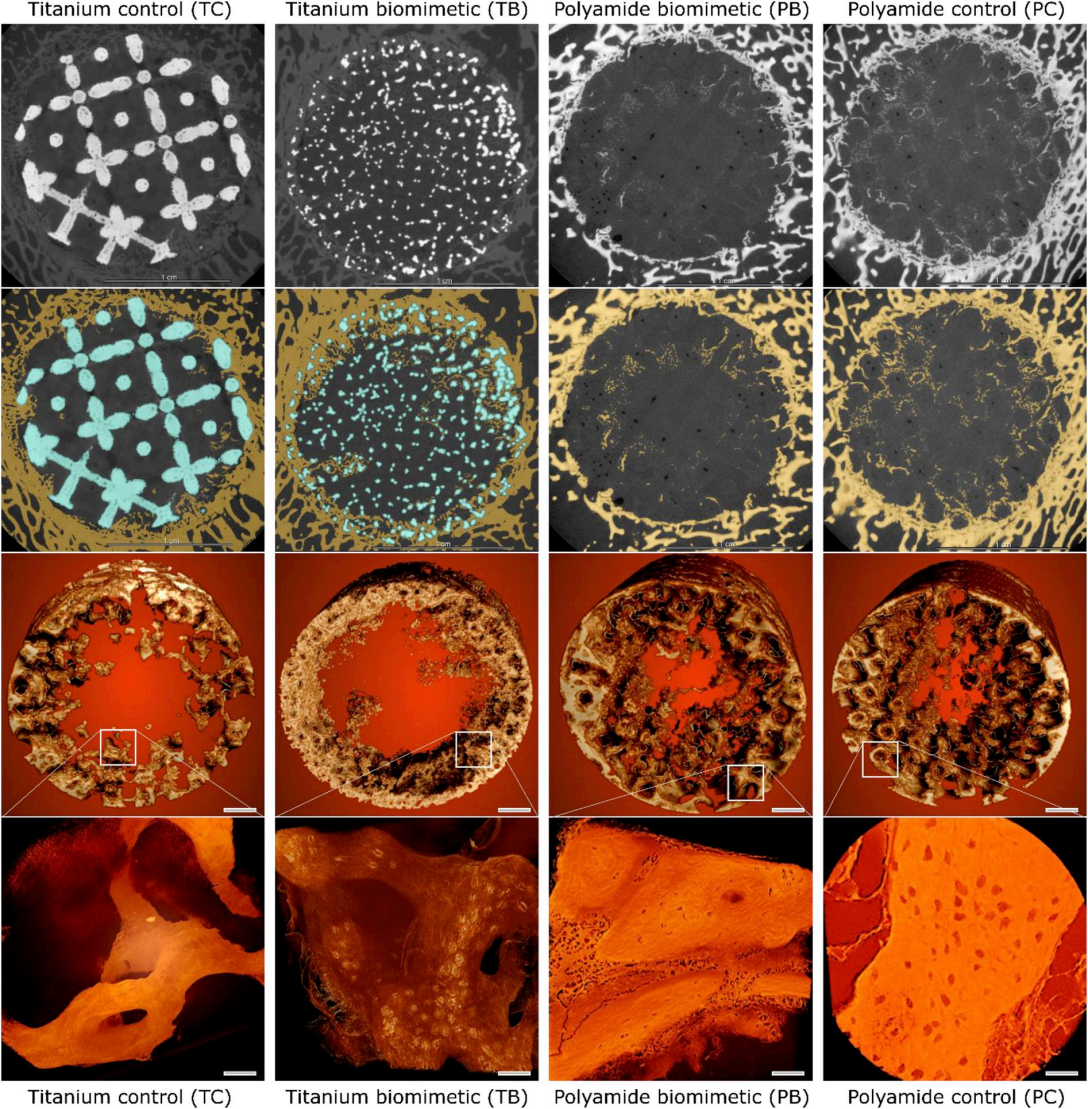
MicroCT images of bone forming within the sheep distal femur defect. Reconstruction of bone ingrowth and segmentation of tomographic images. Test items are grouped in columns, the columns are presented in order of decreasing stiffness (left to right). First row shows 2D digital slices from the middle of the 3D volume, pixel size 30 μm. Second row shows the same slices where bone is labeled in yellow and metal is labeled in blue. Third row shows the ingrown bone within 15 mm cylindrical defects, scaffold elements and bone surrounding the defect are digitally excluded, scale bars are 2 mm. Bottom row shows high-resolution images of the fragments of nascent bone formed within the defects (pixel size 0.5 μm), panels' scale bars are 40 μm. (For interpretation of the references to color in this figure legend, the reader is referred to the Web version of this article.)

We observed a statistically significant difference in bone ingrowth when comparing scaffolds having marked differences in stiffness (TC:PB, TC:PC, TB:PC). The differences in bone ingrowth volume between scaffolds having closer stiffness values (PC:PB, PB:TB, TB:TC) did not reach statistical significance. Volume-percent of bone ingrowth in individual animals is plotted graphically in Fig. 1, for each animal and for each scaffold. The data points from the same animal (*i.e*., right and left legs) are connected with a line. From this, the general trend of increasing bone ingrowth volume with decreasing scaffold stiffness is apparent. In all animals but one, the ingrowth volume was higher in the defect that received a more compliant scaffold.

Three-dimensional μCT rendering of nascent bone within the defect, and high-resolution μCT of ingrown bone elements are shown in Fig. 2. Besides the total volume of the nascent bone, some difference in tissue structure is observed. High-resolution images demonstrate slender osseous elements in both titanium scaffolds (TC and TB, lower panels Fig. 2) whereas more robust formations of bone are present in both polyamide scaffolds (PB and PC, lower panels, Fig. 2).

**Fig. 3:**
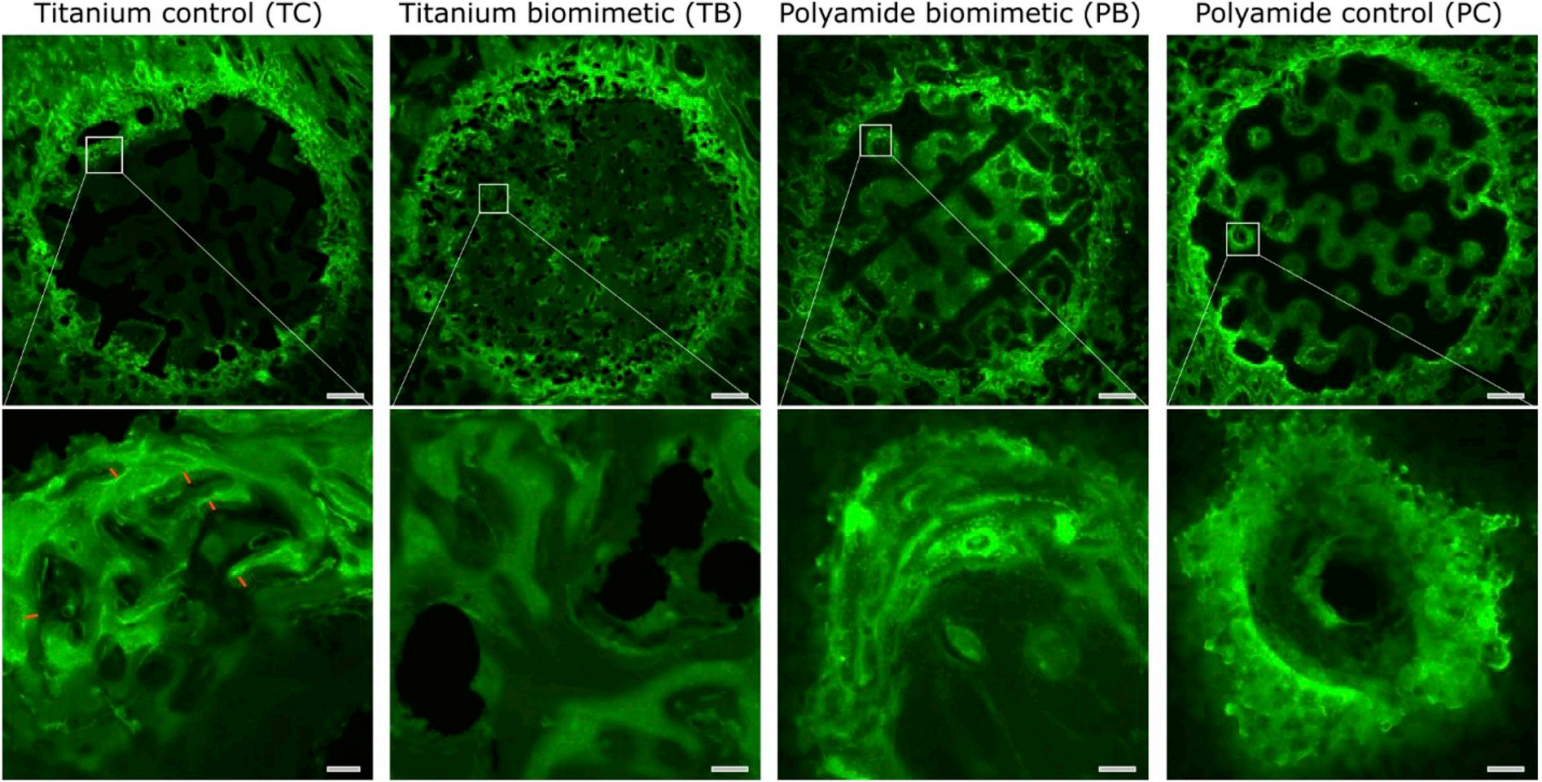
Fluorescence light microscopy of bone formation around, and within sheep distal femur defects. Panels are presented in order of decreasing stiffness, left to right, and show in cross-section the entire scaffold and associated bone (top panels, after multipanel stitching) and a higher magnification image (bottom panels) as indicated (square). The top panels' scale bars are 2 mm and the bottom panels' scale bars are 150 μm. Short orange line markings in the titanium control sample (bottom panel) indicate the amount of nascent bone deposited during the last 14 days of the experiment. (For interpretation of the references to color in this figure legend, the reader is referred to the Web version of this article.)

We used fluorescence microscopy after oxytetracycline injection to investigate osteogenic activity within and surrounding the scaffolds, reflected by the intensity of mineral apposition. Fig. 3 demonstrates variations in binding of the fluorochrome 28 days after implant placement and 14 days prior to animal sacrifice. The brightness of the fluorochrome in sections obtained from the middle of each scaffold (*i.e*., 7–8 mm inwards from the periosteal surface) illustrates high osteogenic activity surrounding all the scaffolds at the periphery of the defect (the bone-implant interface). In comparison, fluorescence within the interior of the scaffolds varied, where differences in osteogenesis are observed between the groups; the lower the scaffold stiffness, the more osteogenic activity is observed within the scaffold interior.

### 3.2 Strong responders and weak responders

Of note, the animals that had the highest net bone gain within the defects, which we refer to as “strong responders” (animals 1, 5–9 and 11), also had the highest difference in bone ingrowth volume between the two different scaffolds implanted (mean difference of 14%, favoring the more compliant scaffold). This is in contrast to values obtained from animals who demonstrated a lower net bone gain, which we refer to as “weak responders” (animals 2–4, 10, 12). The weak responders had a modest difference in ingrowth volume between the two scaffolds each animal received (mean difference of 3%). This division into strong and weak responders can be observed as a varying slope of the lines connecting paired measurements (Fig. 1), where the connected pairs having the higher slope lines are located in the top part of the plot. From this observation, the general trend of bone ingrowth volume can thus be attributed to the data from the strong responders.

**Fig. 4:**
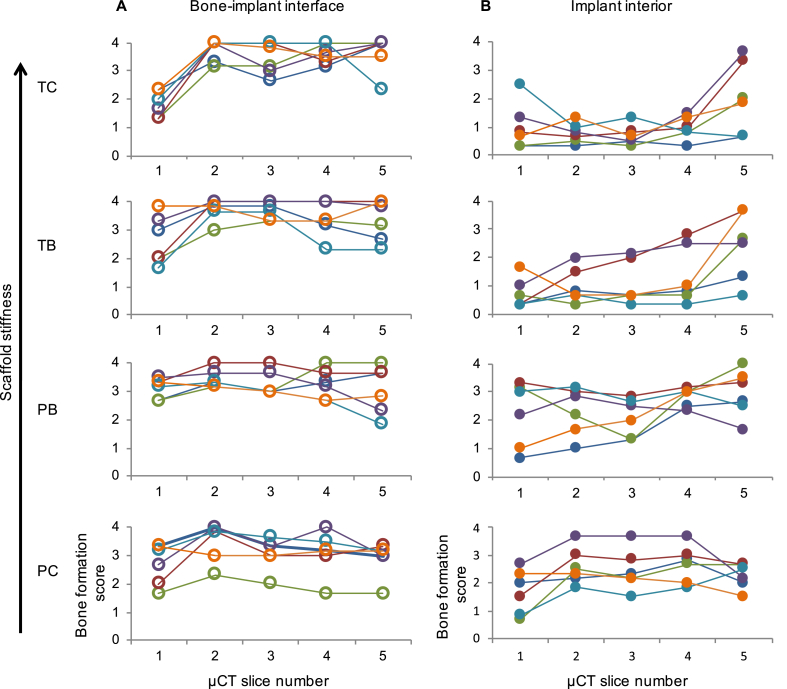
Osteogenic activity at the bone-implant interface (**A**) and implant interior (**B**). Scaffold types are arranged vertically in order of stiffness. Four separate graphs are plotted for the bone implant interface (A) and for the implant interior (B), plotted in order of decreasing scaffold stiffness. For each graph, the micro-CT slice number is shown on the x-axis and the bone formation score is on the y-axis. CT slice 1 was 2 mm from the periosteal surface, while slices 2, 3, 4 and 5 were 5, 8, 11 and 14 mm from the periosteal surface, respectively. On each graph, there are six different color-delineated data points for each CT slice point on the x-axis (though 6 points are not always visible due to overlap as some have similar bone ingrowth). Each color represents one individual scaffold (each scaffold was tested in 6 ovine limbs). Each data point in the graphs represents the mean reported ingrowth from the three observers for that CT slice. The data points for each individual scaffold are linked by colored lines for the five slices. (For interpretation of the references to color in this figure legend, the reader is referred to the Web version of this article.)

To identify the possible mechanism of the individual response heterogeneity, we semi-quantitatively inspected the pattern of bone accretion in strong and weak responders as shown in Fig. 4. We took five virtual μCT slices at different consecutive defect depths, from the periosteal surface towards the bottom of the defect, perpendicular to the implant axis (slice 1, periosteal face of the implant; slice 5, marrow face of the implant; slices 1–5 were evenly spaced by 3 mm). The resultant 120 images (5 from each of 24 samples from 12 animals) were independently scored by three individuals using the criteria in Table 3 and the results are displayed in Table 4 and Supplemental Fig. S4. Semi-quantitative analysis showed that while bone ingrowth at the bone-implant border was comparable and substantial among all the animals, the nascent bone ingrowth within the pores of the scaffolds was highly variable and inversely proportional to the scaffold apparent modulus (Fig. 4, Table 3). For qualitative visual evaluation we plotted the intensity of fluorescence across the sections as a ‘landscape’. Fig. S3 shows that osteogenic activity at the implant-bone interface is comparable in both weak and strong responders (seen as a circular rim of elevated fluorescence intensity). However, osteogenic activity within the implant interior (elevated fluorescence intensity inside the circular rim) is substantial (*i.e*., comparable to the periphery) in the scaffolds of low stiffness in the group of strong responders. This trend was less evident in the weak-responders group (Fig. S3), corroborating Fig. 4 and Table 3.

**Table 3:** Scoring criteria for bone formation at the bone-implant interface (periphery of the defect) and within the interior of the implant and defect.

Score	Bone-implant-interface (periphery of the defect)	Implant interior
0	No observed contact between implant outline and host bone	No bone observed
1	Partial bone contact along approximately one-third of the implant outline	Small specks of high density attributable to bone
2	Partial bone contact along approximately two-thirds of the implant outline	Small fine networks of woven bone or thin deposits on the scaffold struts
3	Complete circular contact between implant outline and host bone	Substantial semilunar deposits of bone within the pores or extensive networks of woven bone
4	Nascent bone engulfs the outer scaffold struts up to fully occupying the outermost tier of scaffold pores	Massive amounts of well-defined bone in all discernible pores of the scaffold

**Table 4:**
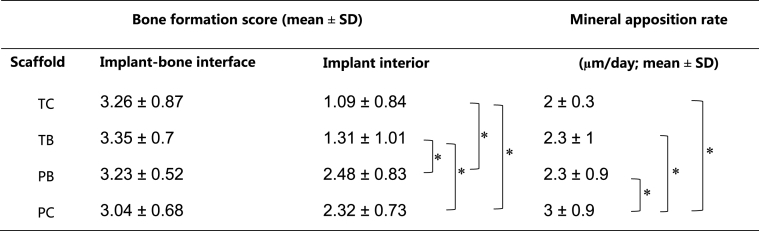
Bone formation score at the implant-bone interface and implant interior, together with the mineral apposition rate for the four scaffolds (from manual measurements of the mineral apposition rate performed from the periphery of fluorescent images). Significant differences (non-paired T-test, *n* = 14, *p* < 0.05) are indicated by brackets. No differences can be identified between the scaffold types in terms of bone regeneration at the bone-implant interface, whereas the score for the amount of nascent bone observed within the interior of both polyamide scaffolds (PB and PC) is significantly higher in comparison to both titanium scaffolds (TB and TC).

### 3.3 Fine structural features of nascent bone

**Fig. 5:**
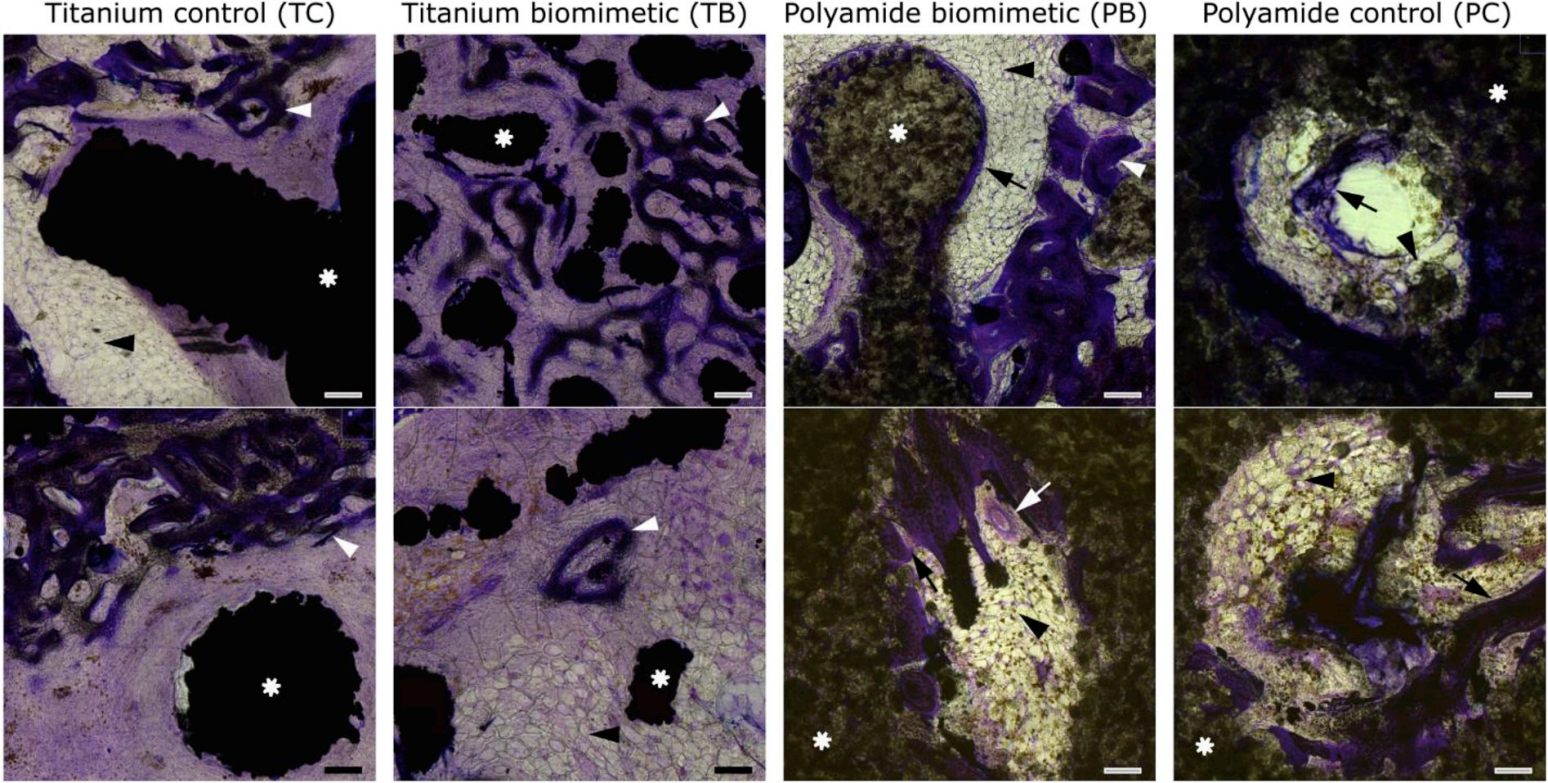
Histology (Paragon staining) of undecalcified sections cut through the center of the scaffolds. Asterisks label the structural elements of the scaffold, which are dark and dense in titanium scaffolds, and more transparent in polyamide scaffolds. White arrowheads indicate woven bone, and black arrows indicate lamellar bone. Formation of lamellar bone is observed only in the polyamide scaffolds PB and PC. Black arrowheads indicate adipose tissue; white arrow in the bottom panel of PB indicates a blood vessel. Panels are presented in order of decreasing stiffness (left to right), all scale bars are 200 μm.

Histological staining of undecalcified sections through the middle of the scaffolds (7 mm deep from the periosteal surface) provides additional information about the pathways of bone formation within the scaffold. Close observation of the nascent bone forming within the two titanium scaffolds (Fig. 5A and B) reveals woven bone tissue, seen as very fine, densely branching struts, forming a delicate mesh that resembles embryonic bone [18]. The most intense formation of fine woven bone is observed at the periphery of the defect, where the host trabecular bone was in contact with the implanted scaffold. The interior of the stiff titanium control scaffolds (TC) is mainly filled with fibrous tissue, occasionally including capillaries. In the case of the titanium biomimetic scaffold (TB), besides intense formation of woven bone at the bone-implant interface, patches of embryonic-looking woven bone can be observed in the center of the defect, seemingly not associated with the surface of the scaffold elements. The networks of woven bone and titanium struts are intercalated without intimate contact and are separated from each other by layers of fibrous tissue.

Nascent bone within the pores of the polyamide scaffolds has a mixed structure; some bone islets formed fine interconnected networks of woven bone, but mostly woven bone elements are sandwiched between layered deposits of lamellar bone, as in Fig. 5 (BP, top panel). Linear deposits of lamellar bone can also be observed directly on the surface of the scaffolds (Fig. 5 PB and PC, top and bottom). Moreover, in many cases of lamellar bone formation directly on the polyamide surface, interlocking contact between mineralized bone and irregular partly-fused grains of polyamide can be observed. In the case of both polyamide scaffolds, clusters of adipocytes, or even areas of adipose tissue of several hundred micrometers wide, can be found associated with nascent bone.

Scanning electron microscopy images obtained with a back-scattered detector (which highlights material density) confirmed the histological findings. Fig. S4 illustrates fine branching networks of woven bone within the titanium scaffolds (Fig. S4 TC and TB). These bone formations contain irregularly shaped osteocytes, characteristic of woven bone [39,40]. In the polyamide scaffold bone formations, brighter woven bone is interposed between surrounding layers of lamellar bone (Fig. S4 PB); the layers of lamellar bone are slightly less bright in the backscattered electron imaging mode, indicating their lower level of mineralization, which is consistent with the literature [41]. For both polyamide scaffolds, interlocking deposits of lamellar bone engulfing the convexities of the sintered polyamide surface are typically observed (Fig. S4 PC).

## 4 Discussion

### 4.1 More strain – more bone?

This study evaluated the relationship between the stiffness of an implanted scaffold and the regenerative osteogenic response of the host, varying both the design and material of the scaffold. The quantitative analysis of bone ingrowth in this study confirmed that generally more compliant scaffolds induced more abundant bone formation in the metaphysis (the correlation was moderate). This has already been shown previously using a long bone shaft model [15,42] or the edentulous part of the ovine mandible which geometrically resembles a long bone shaft [43]. Thus, trabecular bone of an articulating element follows the same biomechanical premise: more strain – more bone. This is also consistent with the concept of stress-shielding, in which a stiff scaffold creates a protected, strain-free niche in the bone that does not encourage osteogenesis [44]. We observed most bone ingrowth within the more-compliant polyamide biomimetic scaffold and the polyamide control scaffold, attributable to stimulation by strain (*i.e*., by high biomechanical demand). However, this general observation has some caveats: the trend of having more bone in response to higher local strains was inhomogeneous both among the experimental animals, and within the implantation site.

### 4.2 Strong and weak responders

Following quantification of bone ingrowth into the scaffolds, the 12 animals used in this study could readily be divided into two groups: strong responders and weak responders. Although all animals were of the same age, sex and breed, some unaccounted factors demonstrably resulted in a different regeneration capacity among the ewes. The inverse correlation between the stiffness of the scaffold and the amount of nascent bone formed indeed can be attributed only to the group of the 7 strong responders, within which the net bone gain was higher, and the difference in bone gain between the implants of different stiffness was more pronounced. In fact, the 5 weak responders – in whom we observed minimal difference in bone gain – contributed to the high variation and scatter of the data. The distinctive difference in bone ingrowth into stiff and compliant scaffolds links together two phenomena – the regenerative potential, and the capacity for mechanosensation. Both mechanosensation capacity and regenerative potential can be explained, among multiple factors, by age [45] and/or by the level of physical activity of the organism [46], although in our study neither the individual levels of mobility of the ewes, nor the group dynamics, were monitored post-operatively. High variation in load exerted on a knee joint has already been reported in sheep [47]; the load exerted on the meniscus and the anterior cruciate ligament is different by up to an order of magnitude across the gait cycle. Interestingly, the inter-subject variation observed in Ref. [47] was deemed representative of human subjects and was attributed to individual gait patterns. The practical implication of this variability for the present study is that bone regeneration cannot be solely linked to implant stiffness, but likely reflects the local biomechanical demands. The biomechanical demand is a product of locally exerted load and material compliance. While the material compliance can be calculated, simulated or tested (more precisely in the case of scaffolds, and approximately in the case of bone), the variance of the locally exerted load is difficult to quantify. For this reason, it would be of great value to consider in the future related studies such aids as local strain monitoring by using strain gauge fitted to the operated limb, and/or mobility monitoring by using wearable external sensors to accrue longitudinal life-style and activity data. Identification of strong and weak responders in the reported group of sheep who are genetically heterogeneous and socially hierarchical is an important piece of information learned that might not be apparent from a small animal experiment.

### 4.3 Two mechanisms for osteogenesis, strain-induced and injury-induced

Evaluation of the osteogenic response separately for the bone-implant interface and for the scaffold interior revealed an unexpected feature: bone regeneration at the interface was independent of both the properties of the implant (whether stiff or compliant) and the regenerative capacity of the host (whether a strong or weak response). In other words, both weak responders and strong responders having either stiff or compliant implants within the defect demonstrated rather similar reactions at the periphery of the osseous defect, with a similar mineral apposition rate of 2–3 μm/day and noticeable osseointegration (host bone engulfing peripheral elements of the scaffold). On the other hand, osteogenesis within the interior of the scaffold depended on two parameters, the scaffold's apparent modulus and the regenerative potential of the host. This dual response (peripheral and central, with respect to the implant) seemingly illustrates two pathways of bone formation, one being the reaction to the local effect of inflammation and/or trauma, and the other being the reaction to local strain. Of note, while 3D quantification of bone ingrowth in the entire scaffold (i.e., both periphery and interior) produced insignificant differences in scaffolds with close values of apparent moduli, the scoring of nascent bone formation within the scaffold interior did yield a statistically significant difference between PB and TB. Therefore, there is a possibility that the significance of the difference in pairs with close modulus values was blurred by the peripheral, injury-dependent response that was uniform. We observed mainly woven bone formation at the bone-implant interface, and a mixture of different proportions of woven and lamellar bone within the scaffold interior. Indeed, a study by Turner et al. [48] showed that woven bone appears in response to local irritation and does not depend on strain levels, whereas lamellar bone is deposited in response to strain exceeding about 0.1%. McBride et al. [49] define an “injury response” (woven bone formation) and an “adaptive response” (mostly lamellar bone formation), which is consistent with the findings of our study, where we observed the injury response at the periphery of the defect at the implant-bone interface, and the adaptive strain-dependent response in the interior of the scaffold. In practical terms, the injury response at the bone-scaffold border is “guaranteed”, whereas the strain-induced bone formation in the scaffold interior is conditioned by the local biomechanical demand and is as (un)certain as the host's regenerative response is. Thus, careful consideration of these two factors, both the adequacy of scaffold's stiffness/compliance and the host responsiveness, leads to a critical question: whether a degradable scaffold is an indisputably ideal solution for bone regeneration. While it is definitely desirable to achieve a complete replacement of the graft with the host tissue, the reality may impose certain constraints, such as the mechanosensitivity or regenerative capacity of the host. Undoubtedly, longer studies are essential to clarify whether a “weak responder” may eventually catch up with a “strong responder” in terms of the strain-dependent bone formation, or whether the weak response can be augmented by supplementary physical or pharmaceutical stimuli. Perhaps in some cases of uncertain regenerative potential of the host due to inherently low metabolic activity, old age, frailty, or low level of physical activity, a fail-safe strategy of nondegradable or partially-degradable graft design could be pursued even when a small volume of bone has to be reconstructed.

### 4.4 Woven and lamellar bone pathways

Woven bone is different from lamellar bone in terms of structure and function [50]. A common notion is that woven bone is initiated by higher strains than lamellar bone [49] and it is an injury response [51]. However, woven bone normally precedes lamellar bone formation for the reason of providing anchorage and a suitable substrate for lamellar bone deposition, be it in the scenario of damage repair [52] or normal fetal development [53]. Woven bone formation advantageously takes place *de novo*. Although its organization and mechanical properties are inferior to lamellar bone [22], woven bone provides a transient biological scaffold whose mechanical properties can be further adapted and fine-tuned by surface deposition of lamellar bone [39]. Thus, the presence of woven bone is not only an indicator of high local strains, but it is also a trait of the initial, transient stage of osteogenesis. In this study, we observed vast networks of woven bone within the interior of the TB scaffold. These woven bone formations were not associated with the surface of titanium scaffold struts. This is consistent with the description of woven bone forming within a fracture callus and not being associated with the surface of existing trabeculae, referred to as “true osteoinduction” [54]. Within the more compliant PB and PC scaffolds, where strain was higher, at the time of sample retrieval, woven bone had mostly given way to lamellar bone: deposits of lamellar bone overlaid onto reticulate elements of woven bone.

The question as to whether the physiological woven-to-lamellar bone transition is accelerated by higher biomechanical demand, or decelerated within a low-strain niche, can be answered by comparing the localization of mineral-binding fluorescent label in undemineralized sections. We administered the fluorescent label 2 weeks prior to sample collection; the observation of fluorescent reticulate elements of exclusively woven bone within the titanium scaffold interiors indicates that low strain may decelerate the normal sequence of osteogenic events (*i.e*., when woven bone within a low-strain niche was not eventually superimposed by lamellar bone, as would normally occur).

### 4.5 Polyamide serves as a woven-bone surrogate substrate for lamellar bone

The nascent bone tissue that we observed within the polyamide scaffolds (PB and PC) displayed more mature characteristics, such as having lamellar structure and co-alignment of osteocytes. Polyamide scaffold struts and nascent bone were in close interlocking contact, with mineralized bone formation observed around the scaffold elements (each about 1 mm in diameter) and around partially fused, individual grains of polyamide substrate (each about 50 μm in diameter). The close contact between the bone and scaffold material can be explained by the similar values of material bone and polyamide elastic moduli (being in the range of several GPa), so that osteoprogenitor cells and osteoblasts populate the polyamide scaffold surface in a manner similar to bone surfaces during normal development [55]. Following an established definition [21], *de novo* bone formation by newly-differentiated osteogenic cells within nonmineralized tissue is called osteoinduction, whereas bone deposition on an existing surface/substrate is called osteoconduction. The surface required for osteoconduction can be natural (such as woven bone) or artificial (such as an implanted material). The desired outcome of bone tissue engineering is the formation of mature, mechanically superior and adaptive lamellar bone, rather than the more delicate and primitive woven bone. For these reasons, provision of a nonresorbable, suitably compliant surrogate construct for direct lamellar bone apposition (*i.e.*, osteoconduction within an artificial scaffold) could be a viable strategy in bone tissue engineering.

### 4.6 A note on observations of adipose tissue found in defect sites: a proxy for bone maturation

The compliant scaffolds showed a more advanced stage of bone formation, where lamellar bone partly overlaid the woven bone. The presence of adipocytes, either in small clusters, or as vast areas, accompanied the appearance of mature lamellar bone in the case of the polyamide scaffolds. Indeed, bone, fibrous stroma and adipocytes are the progeny of marrow pericytes, also known as skeletal stem cells (SSCs) [56]. Adipocytes are large, roughly spherical or ellipsoid cells containing a unilocular lipid droplet, and are highly recognizable in histological sections. Of all the derivatives of SSCs, adipocytes appear chronologically last, when committed osteoprogenitors establish their association with blood vessels [56]. Marrow adipocytes conduct the function of reversible microvasculature pruning; they accumulate lipids to swell at the outer surface of marrow sinusoids, and thus precisely regulate microcirculation and hematopoietic progenitor traffic [57]. The presence of adipocytes associated with nascent bone deposits within the compliant scaffold interior could be a potential indicator of an advanced stage of bone development and maturation.

### 4.7 Conclusions and outlook

The results of this 6-week study in sheep confirmed the previously observed phenomenon of more intensive osteogenesis in response to mechanical challenge. Interestingly, it also produced new questions that entail future work. For example, what is the relationship between reparative bone formation and host regenerative capacity, what are the relative proportions of injury- and strain-driven osteogenesis, and what is the pace of the woven-to-lamellar bone transition? A longer-term study would benefit from (*i*) incorporating *in vivo* strain measurements to determine the extent of the “biomechanical demand” and its relation to osteogenesis, (*ii*) monitoring group dynamics and locomotion, for studying variations in individual physical activity and gait patterns, (*iii*) administration of multiple fluorescent labels to better quantify bone formation over longer times in the context of osteoinduction and osteoconduction, (*iv*) injection of x-ray contrast agent prior to euthanasia for comparison of microvascularization of various implants, and finally, (*v*) a larger metaphyseal defect could be considered, with variable stresses and strains at its interfaces, enabling load-specific responses. These refinements will serve a better platform for tuning the mechanical properties of 3D-printed biomimetic scaffolds, for the pressing clinical demands of substantial bone defects. The multidimensional Goldilocks’ principle of “not too much and not too little” may thus be addressed by flexing both the design and material choice to suit to the widely varying mechanical demands of reconstructive surgery.

## Author contributions

Study design: NR, ORB and MMS. Study conduct: NR, ORB, SG. Data collection: NR, ORB, SG, AEW, LC. Data analysis: NR, ORB and SG. Data interpretation: NR and MMS. Drafting manuscript: NR, ORB, SG. Revising manuscript content: NR, ORB, SG, AEW, LC, GWB, JJ, JPC and MMS. Approving final version of manuscript: NR, ORB, SG, AEW, LC, GWB, JJ, JPC and MMS.

## Conflicts of interest

None.

## Non-authors

We thank Imperial College London FILM facility and Stephen Rothery for light microscopy imaging (training and assistance); Dee Fischer (Embody) for design troubleshooting for NR and printing of the PA scaffolds; Pamela G. Robey (NIH) for insightful discussions of bone histological sections.

## Data and materials availability

Raw data is available upon request from rdm-enquiries@imperial.ac.uk.
