# Supplementary material for: Individual response variations in scaffold-guided bone regeneration are determined by independent strain- and injury-induced mechanisms
Source: Biomaterials. 2019 Feb;194:183–94. doi: 10.1016/j.biomaterials.2018.11.026 (PMC6345626; doi:10.1016/j.biomaterials.2018.11.026)
Supplement: Multimedia component 1 [file mmc1.docx]

**Supplementary materials**


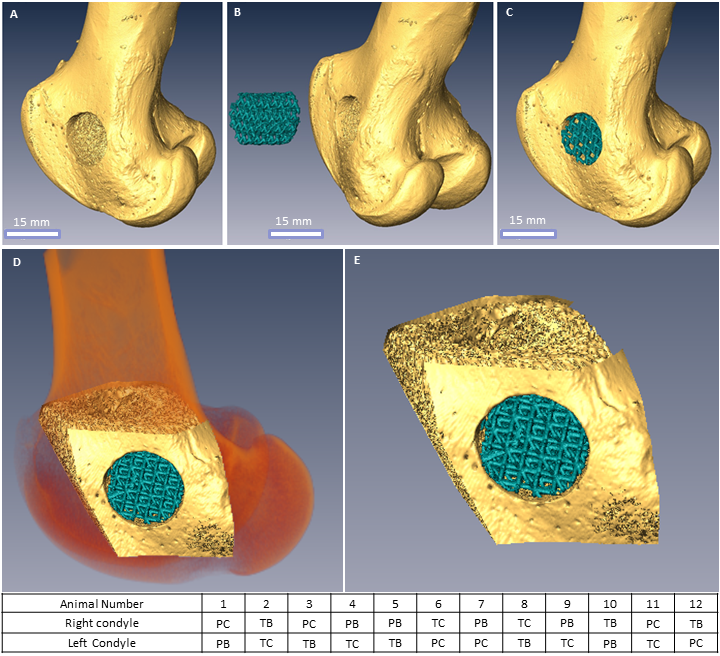


**Fig. S1** Schematic of surgical scaffold placement, specimen retrieval and test item assignment among the animals.

**
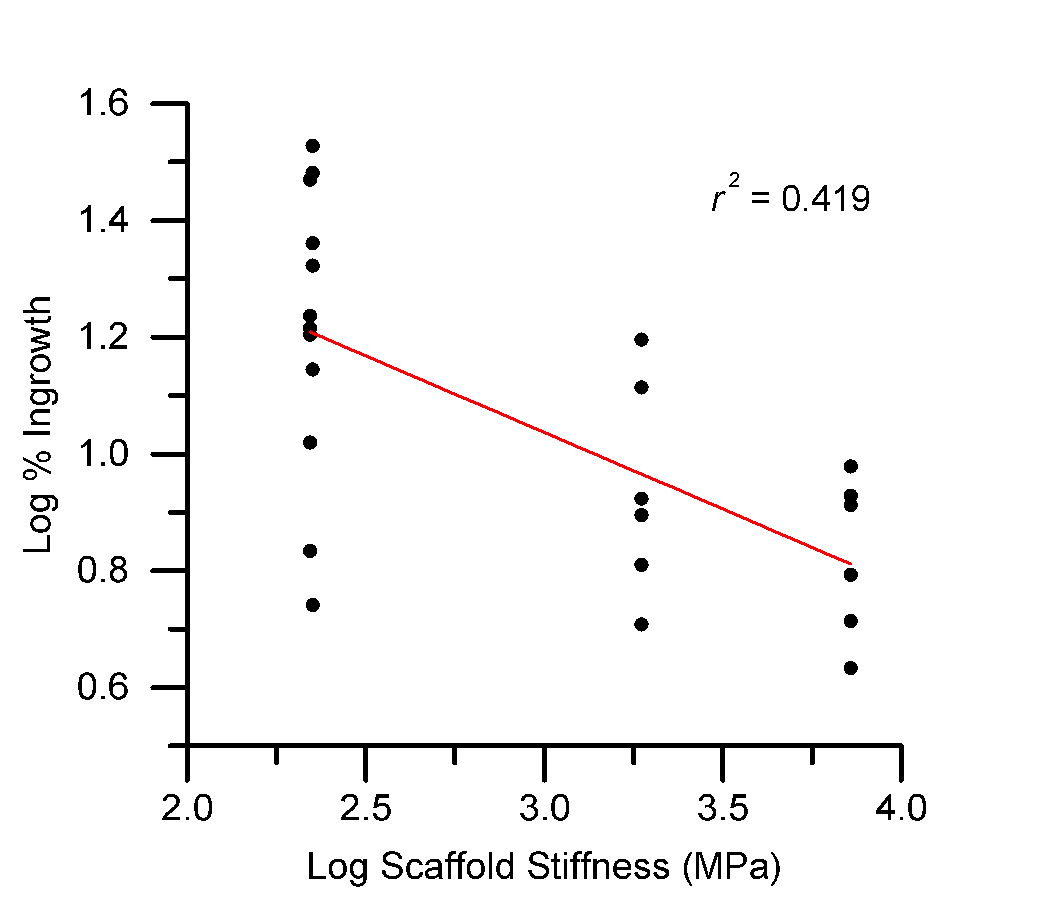
**

**Figure S2.** Logarithmic regression of bone ingrowth versus scaffold stiffness. The scaffold apparent modulus, in MPa and after log transformation, is plotted on the x-axis. As the apparent modulus of the nylon scaffolds is similar they appear as the same number after log transformation, but are in fact, slightly different (2.34 and 2.35 MPa). The log-transformed percentage bone ingrowth is plotted on the y-axis. Pearson’s regression line is plotted on the graph, showing a moderate inverse correlation between scaffold stiffness and bone ingrowth (*r*^2^ = 0.419, *p* = 0.001).

**Figure S3**. Fluorescence intensity landscape of bone formation around, and within, metaphyseal defects. Three-dimensional plots showing relative intensity of osteogenesis at the implant-bone interface and within the interior of the defect as a function of scaffold stiffness and of the host response. Whereas the intensity of osteogenesis is substantially elevated at the interface in all plots, a considerably elevated landscape is observed in the defect interior of only the polyamide scaffolds PB and PC, and only in the group of strong responders. Somewhat elevated intensity is also observed in the compliant polyamide control scaffold interior (PC, weak responder) and in the biomimetic titanium scaffold interior (TB, strong responder). Scale bar 5 mm.


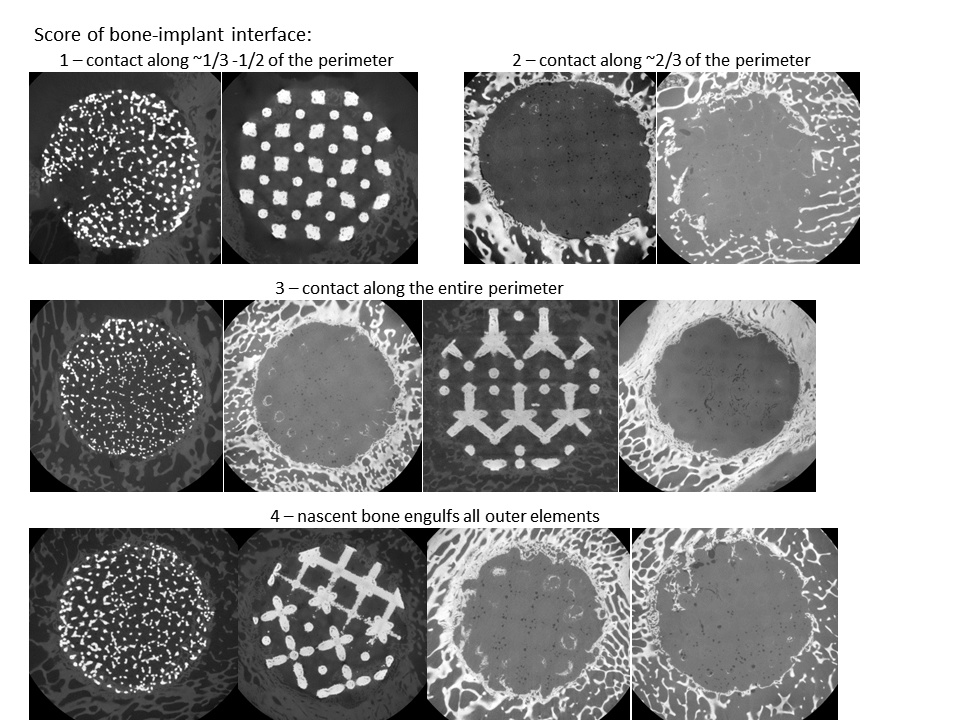

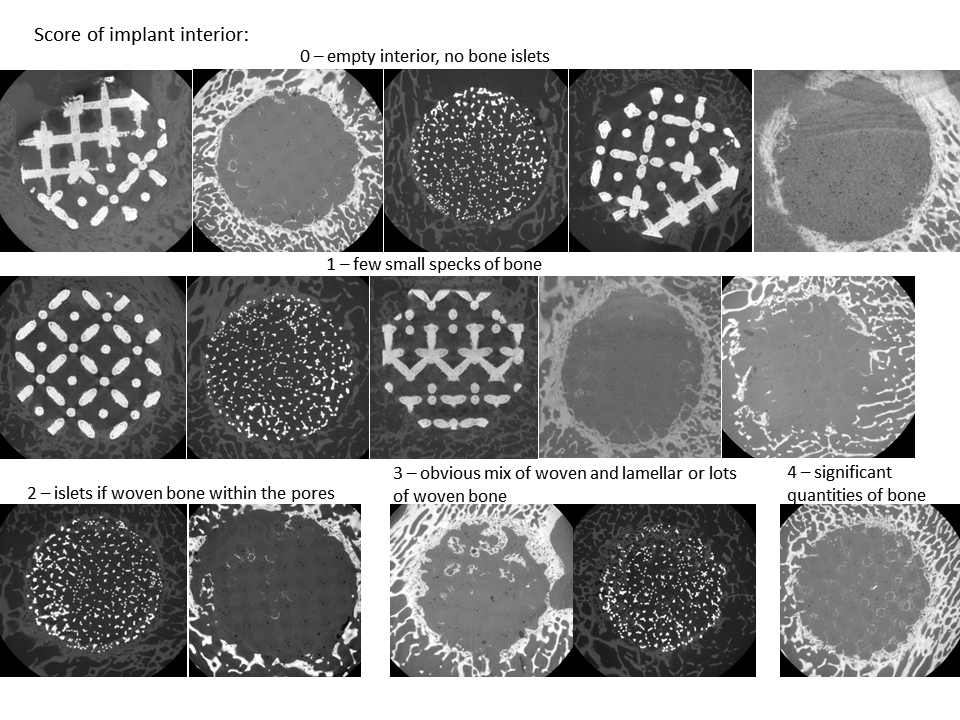


**Figure S4.** Visual guidelines for scoring bone ingrowth on 2D µCT slices, as per Table 4.


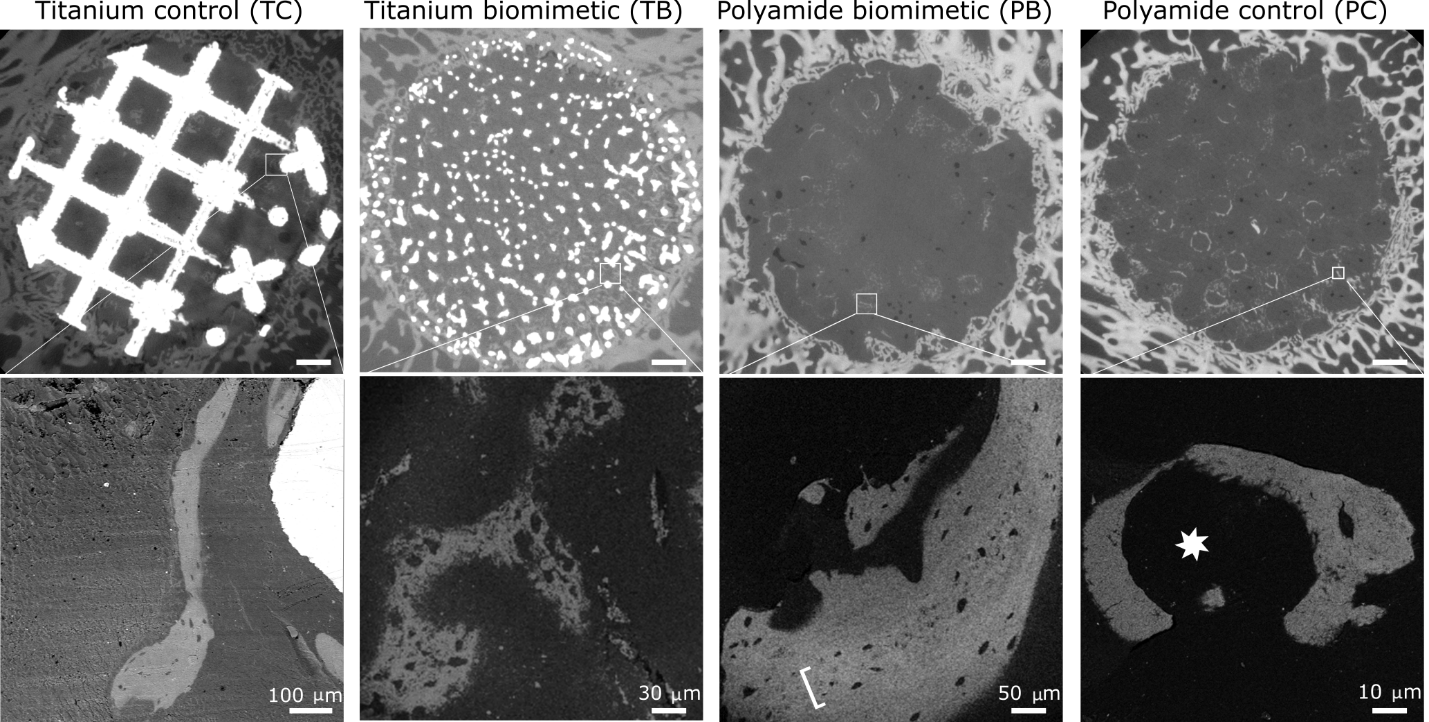


**Figure S5**. Images of nascent bone within the scaffold interior. In each pair of images assembled vertically, the panel on the top is an overview µCT slice, and the panel on the bottom is a scanning electron micrograph (backscattered electron imaging). White bracket in polyamide biomimetic scaffold (bottom) indicates a woven bone core with apposition of lamellar bone. Asterisk in polyamide control (bottom) indicates a polyamide grain partly fused with the bulk of the scaffold strut. Panels are presented in order of decreasing stiffness (left to right), the top panels’ scale bars are 2 mm.
